# Caveolin-1 expression is elevated in claudin-low mammary tumor cells

**DOI:** 10.1186/1475-2867-12-6

**Published:** 2012-02-22

**Authors:** Devan E Thompson, Megan D Siwicky, Roger A Moorehead

**Affiliations:** 1 Department of Biomedical Sciences, Ontario Veterinary College, University of Guelph, 50 Stone Road East, Guelph, ON, Canada N1G2W1

## Abstract

**Background:**

Caveolin-1 is a scaffolding protein found in plasma membrane invaginations known as caveolae. Caveolin-1 can regulate a number of intracellular processes such as signal transduction, cholesterol metabolism and vesicular transport. With respect to breast cancer caveolin-1 has been observed in both tumor cells and stromal cells surrounding tumors however most of the recent research has focused on how the loss of caveolin-1 in the stromal cells surrounding the tumor alters the tumor microenvironment.

**Methods:**

Caveolin-1 expression was evaluated in (1) mammary tumors induced by the transgenic overexpression of the type I insulin-like growth factor receptor (IGF-IR), (2) mammary tumors that became independent of IGF-IR signalling and acquired a claudin-low genotype, (3) two murine mammary epithelial tumor cell lines and (4) two murine mammary claudin-low tumor cell lines.

**Results:**

We found that mammary tumors induced by IGF-IR overexpression expressed low levels of caveolin-1 while mammary tumors that became independent of IGF-IR signalling expressed considerably higher levels of caveolin-1. Interestingly, pockets of caveolin-1 positive cells could be observed in some of the IGF-IR-induced mammary tumors and these caveolin-1 positive cells were associated with tumor cells that expressed basal cytokeratins (cytokeratins 5 and 14). This caveolin-1 expression pattern was maintained in the murine mammary tumor cell lines in that the epithelial mammary tumor cell lines expressed little or no caveolin-1 while the claudin-low cell lines expressed caveolin-1.

**Conclusions:**

Our model indicates that mammary tumor cells with epithelial characteristics lack caveolin-1 while mesenchymal tumor cells express caveolin-1 suggesting that caveolin-1 may serve as a marker of mammary tumor cells with mesenchymal characteristics such as claudin-low breast tumors.

## Background

Caveolin-1 (Cav-1) is a member of the caveolin family which consists of Cav-1, Cav-2 and Cav-3. Caveolins function as scaffolding proteins and are found in plasma membrane invaginations known as caveolae. Cav-1 and Cav-2 are co-expressed and found in a number of cell types including endothelial cells, adipocytes, and type I alveolar cells while Cav-3 is expressed in skeletal, cardiac and smooth muscle cells [1-4]. In normal cells caveolins regulate signal transduction, cholesterol metabolism, and vesicular transport.

Most of the work performed on Cav-1 in breast cancer has been directed towards the stromal cells. Loss of Cav-1 in stromal cells surrounding breast cancers has been associated with disease recurrence, metastasis, tamoxifen resistance and poor clinical outcome [5,6]. It has been suggested that loss of Cav-1 in tumor-associated fibroblasts induces aerobic glycolysis in these cells resulting in increased production of pyruvate and lactate. The adjacent tumor cells would then utilize these substrates for ATP production. This phenomenon has been termed "Reverse Warburg Effect" [7-10]. A modification of this hypothesis suggests that loss of stroma Cav-1 increases autophagy in the tumor microenvironment leading to recycled nutrients that can be utilized by the tumor cells and protection of the tumor cells from apoptosis and autophagy [11-14].

There have been some studies investigating Cav-1 levels in breast tumor cells. In two independent studies Cav-1 expression was found to be lower in human breast cancer cell lines than normal mammary tissue [15,16]. In addition, overexpression of Cav-1 in human breast cancer cell lines inhibited proliferation and soft agar colony formation [16]. However, studies by Heiser et al. [17] and Elsheikh et al. [18] showed that Cav-1 was frequently observed in basal but not luminal, breast cancer cells. Therefore, Cav-1 may be expressed only in certain breast cancer subtypes.

Cav-1 null mice have been created and display mammary epithelial hyperplasia [19-21]. Since Cav-1 expression is lost in all cells in these mice it is difficult to determine whether the hyperplasia results from Cav-1 loss in the epithelial cells, stromal cells or both. To examine the effects of Cav-1 loss directly on mammary epithelial cells, mammary epithelial cells from Cav-1 null mice were transplanted into the mammary fat pad of wild type mice. Cav-1 deficient mammary epithelial cells displayed increases in proliferation, terminal end bud area, and mammary ductal thickness [21].

Our group became interested in Cav-1 following the analysis of DNA microarray results that compared mammary tissue from wild type mice to tumor tissue from transgenic mice that express elevated levels of the type I insulin-like growth factor receptor (IGF-IR) in mammary epithelial cells. Cav-1 was significantly downregulated (~37-fold) in the mammary tumor tissue compared to normal mammary tissue [22]. Interestingly, tumors that recurred following IGF-IR downregulation in the mammary tumors acquired a claudin-low phenotype and genotype and expressed Cav-1 at significantly higher levels (~14-fold) than the initial mammary tumor [22]. To study these differences further, Cav-1 expression was evaluated in mammary tumor tissue and mammary tumor cell lines. We found that the differences in Cav-1 expression were a result of tumor cell Cav-1 rather than stromal Cav-1. Epithelial mammary tumors expressed low levels of Cav-1 while claudin-low mammary tumors expressed much higher levels of Cav-1. These findings were confirmed in two murine mammary epithelial tumor cell lines and two murine mammary claudin-low cell lines in that the claudin-low cell lines expressed elevated levels of Cav-1 compared to the epithelial ones. Therefore, our findings suggest that Cav-1 expression can be used to differentiate mammary tumors with epithelial characteristic from those with mesenchymal or more specifically, claudin-low characteristics.

## Methods

### MTB-IGFIR transgenic mice

The initial generation and characterization of transgenic mice overexpressing the type I insulin-like growth receptor (IGF-IR) has been presented in [23]. Generation of tumors following downregulation of the IGF-IR transgene has been described in [24]. Animals were housed and cared for following guidelines established by the Central Animal Facility at the University of Guelph and the guidelines established by the Canadian Council of Animal Care.

### Cell lines and culture conditions

4T1 cells were obtained from the ATCC (Manassas, VA) and were cultured in DMEM media containing 10% FBS, 1 mM sodium pyruvate, 10 mM Hepes and 4 mM L-glutamine. RM11A, RJ345 and RJ348 cells were created from different tumors of MTB-IGFIR transgenic mice and they are described in [25]. RM11A, RJ345 and RJ348 cells were cultured in media described in [26] with RM11A and RJ345 being cultured in media supplemented with 10 μg/mL doxycycline for transgene induction while RJ348 cells were cultured in doxycycline-free media.

### Western blotting

Western blotting was performed as previously described [23]. Caveolin-1 antibody was obtained from BD Biosciences (Mississauga, ON, Canada) and used at a 1:20,000 dilution while the β-actin antibody was purchased from Sigma (St Louis, MO) and used at a 1:2,000 dilution. Secondary antibodies were purchased from Cell Signaling Technology (Danvers, MA) and used at a 1:2,000 dilution. Bands were visualized using a FluorChem9900 imaging system and AlphaEaseFC software (Alpha Innotech, San Leandro, CA).

### Immunohistochemistry

Immunohistochemistry was performed as previously described [23]. Caveolin-1 antibody (BD Biosciences, Mississauga, ON, Canada) was used at a 1:10,000 dilution while cytokeratin 5 and cytokeratin 14 antibodies were purchased from Abcam (Cambridge, MA) and used at a 1:100 dilution. Primary antibodies were detected with the appropriate secondary antibody (Sigma, St Louis, MO) used at a 1:200 dilution and Sigma Fast 3,3'-diaminobenzidine tablets (Sigma, St Louis, MO).

### Immunofluorescence

Immunofluorescence was performed as previously described [26]. Caveolin-1 antibody (BD Biosciences, Mississauga, ON, Canada) was used at a dilution of 1:10,000. Cell nuclei were counterstained with DAPI. Images were captured on an Olympus BX61 fluorescent microscope (Olympus, Center Valley, PA) using Metamorph imaging software (Molecular Devices, Sunnyvale, CA).

### Quantitative real-time PCR

Total RNA was extracted from cells cultured to approximately 75% confluency, 2 d after plating using the Ambion *mir*VANA miRNA isolation kit (Applied Biosystems, Streetsville, ON), in accordance with the manufacturer's instructions (without the miRNA enrichment step). Real time PCR was performed as described in [24]. All primers were obtained from Origene (Rockville, MD).

### Statistics

Statistical significance was determined using an ANOVA followed by a Tukey's HSD post-hoc test. Values were considered statistically significant when *p *< 0.05.

## Results

DNA microarray analysis was used to compare three sets of murine tissues; (i) adult, wild type mammary tissue (WT), (ii) primary mammary tumors induced by transgenic IGF-IR overexpression which present with an epithelial morphology (PMT; primary mammary tumor), and (iii) mammary tumors that resume growth following IGF-IR downregulation. These tumors that arise following IGF-IR downregulation express low levels of the IGF-IR and present with a spindle shaped morphology (RST; recurrent spindle tumor). The partial characterization of these tumors has been presented in [24] while characterization of the IGF-IR transgenic mice (MTB-IGFIR mice) has been presented in [23].

**Figure 1:**
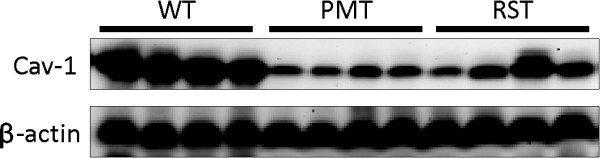
**Western blot of Cav-1 protein in 4 independent wild type (WT) mammary tissue sample, 4 independent primary mammary tumor (PMT) tissue samples induced by transgenic overexpression of IGF-IR, and 4 independent recurrent spindle tumor (RST) tissue samples which developed after the downregulation of the IGF-IR transgene**. β-actin served as a loading control

The DNA microarray analysis indicated that Cav-1 was reduced approximately 37-fold in the PMT tissue compared to WT mammary tissue [22]. As Cav1 is expressed in adipocytes, endothelial cells, myoepithelial cells and fibroblasts but not epithelial cells [1-4] this finding was not completely surprising as the PMTs are composed primarily of epithelial cells [23]. Interestingly, Cav-1 expression was somewhat restored in the RST tissue. Cav-1 expression as determined by the DNA microarray was approximately 14-fold higher in the RST samples compared to the PMT samples [22]. To confirm the DNA microarray findings, western blotting was performed and these blots showed that the levels of Cav-1 protein were highest in normal mammary tissue from WT mice, reduced in the PMTs and somewhat restored in the RSTs (Figure 1).

**Figure 2:**
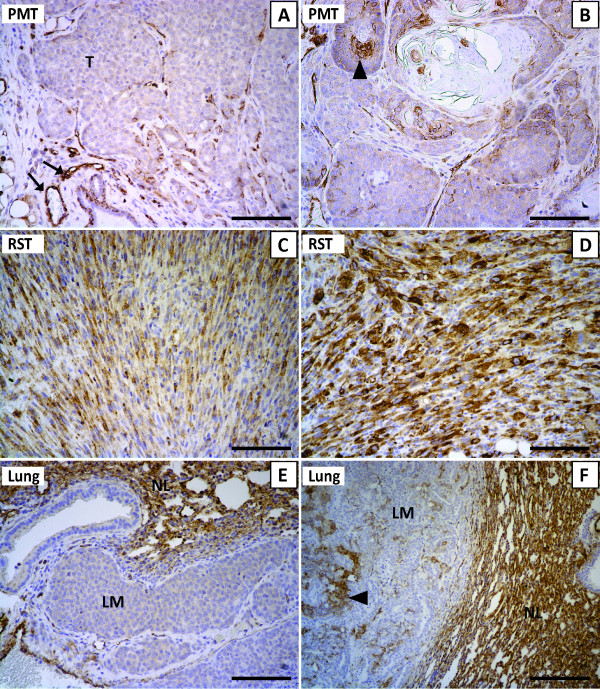
**Cav-1 immunohistochemistry in PMT tissue (A, B), RST tissue (C, D) and two different lung metastases (E, F)**. The arrows in (A) indicate Cav-1 positive cells lining blood vessels in normal mammary tissue while T marks the mammary tumor. The arrowhead in (B) highlights Cav-1 positive tumor cells within a PMT sample. In panels (E, F) LM identifies the lung metastasis while NL identifies normal lung tissue. Scale bars, 100 μm

To determine whether Cav-1 was being expressed in the tumor cells and/or the stromal cells, immunohistochemistry for Cav-1 was performed. In the PMT tissue sections adipocytes and cells lining blood vessels stained strongly for Cav-1 protein as did basal cells in normal mammary ducts (Figure 2A; arrow). The tumor cells themselves primarily expressed only low levels of Cav-1 protein (Figure 2A; T) however some regions of the PMT had clusters of cells that stained positive for Cav-1 (Figure 2B, arrowhead). Therefore, most of the tumor cells in PMTs express low levels of Cav-1 however, pockets of tumor cells expressing high levels of Cav-1 can be found within some of these tumors. PMTs in the MTB-IGFIR transgenic mice are not typically surrounded by fibroblasts. In a few tumors where fibroblasts could be found adjacent to the mammary tumor, these fibroblasts displayed little or no staining for Cav-1 (Figure 2A, B). In the RST tissue sections, Cav-1 protein could be detected at moderate (Figure 2C) or high (Figure 2D) levels in the tumor cells themselves. The level of Cav-1 staining was variable between different RSTs and within individual RSTs. Fibroblasts associated with RSTs could not be readily detected and thus the level of Cav-1 in RST associated fibroblasts could not be determined. The levels of Cav-1 were also examined in lung metastases that developed in the MTB-IGFIR transgenic mice. Approximately 30% of MTB-IGFIR transgenic mice develop lung metastases. In general, the lung metastases expressed very low levels of Cav-1 compared to the surround lung tissue (Figure 2E). One mouse had a number of large lung metastases and these metastases had pockets of Cav-1 positive cells (Figure 2F, arrowhead).

**Figure 3:**
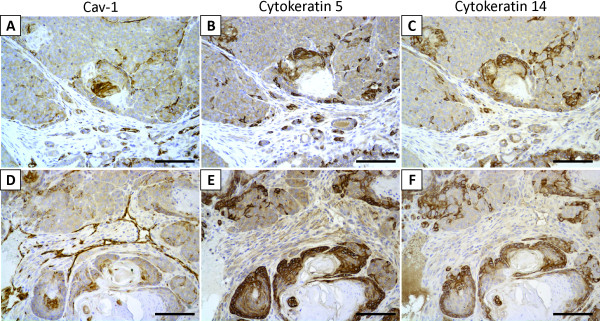
**Immunohistochemistry for Cav-1 (A, D), cytokeratin 5 (B, E), and cytokeratin 14 (C, F) on serial sections from two different PMT samples**. Scale bars, 100 μm

The pockets of Cav-1 positive cells observed in the PMT samples appeared to be associated with regions that had undergone some squamous differentiation. These regions were reported previously [23] and were shown to contain cells expressing cytokeratins 5 and 14. To determine whether Cav-1 positive cells also expressed cytokeratin 5 and 14, immunohistochemistry for Cav-1, cytokeratin 5 and cytokeratin 14 was performed on serial sections of two distinct mammary tumors. Although Cav-1 failed to completely co-localize with either cytokeratin 5 or cytokeratin 14, most of the Cav-1 positive cells were found in regions of the tumor were there was an abundance of cytokeratin 5 or cytokeratin 14 positive cells (Figure 3). Little or no Cav-1 was observed in regions of the tumor where the cells were negative for either cytokeratin 5 or 14. This suggests that Cav-1 is expressed at low levels in tumor epithelial cells but at higher levels in regions associated with basal/mesenchymal tumor cells.

**Figure 4:**
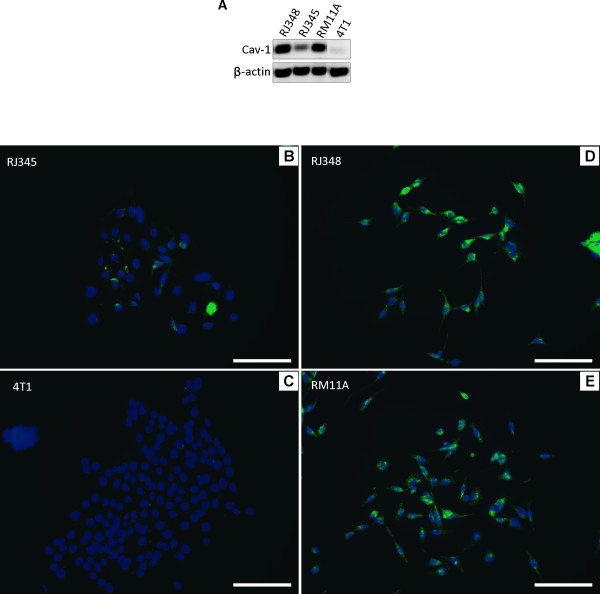
**(A) Western blot of Cav-1 protein in two epithelial murine mammary tumor cell lines (4T1 and RJ345) and two murine mammary claudin-low cell lines (RJ348 and RM11A)**. β-actin served as a loading control. Immunofluorescence of Cav-1 (green) in RJ345 (**B**), 4T1 (**C**), RJ348 (**D**) and RM11A (**E**). Blue fluorescence indicates cell nuclei staining positive for DAPI; scale bars, 50 μm

Several cell lines have been established from the PMT and RST tumors of the MTB-IGFIR transgenic mice. These lines have recently been characterized [25]. Two of the cell lines (RM11A and RJ348) have characteristics of claudin-low mammary tumors while a cell line, RJ345 has characteristics similar to luminal tumors [25]. 4T1 cells are a commercially available murine mammary epithelial cell line [27] that has previously been shown to have characteristics similar to the RJ345 cells [25]. Western blotting and immunofluorescence for Cav-1 protein and qRT-PCR for Cav-1 mRNA revealed that tumor cells themselves can express Cav-1 and the claudin-low mammary tumor cell lines (RJ348 and RM11A) expressed higher levels of Cav-1 protein and mRNA than the epithelial tumor cell lines (Figure 4 and Table 1).

**Table 1:** Cav-1 mRNA Levels

Cell Line	Cav-1 mRNA
4T1	0.12 ± 0.02^1^
RJ345	0.37 ± 0.08
RJ348	1.81 ± 0.06^a^
RM11A	1.06 ± 0.20^a^

^1^Values represent mean ± SEM and are Expressed Relative to HPRT mRNA

^a^Significantly higher than 4T1 or RJ345

## Discussion

Most of the current research on Cav-1 in breast cancer has demonstrated that loss of Cav-1 expression in the stromal cells surrounding the tumor is associated with early tumor recurrence and poor clinical outcome [5,6]. It has been proposed that loss of stromal Cav-1 induces oxidative stress within the fibroblasts which in turn results in an increased secretion of lactate and pyruvate by the stromal fibroblast and/or autophagy in the stromal compartment [13,14,28]. In both instances, the stromal cells provide essential fuel for tumor growth. Loss of stromal Cav-1 has also been associated with epithelial to mesenchymal transition (EMT) of mammary epithelial cells. It was shown that conditioned media from Cav-1 null mammary stromal fibroblasts could induce EMT of normal mammary epithelial cells [29]. EMT is a program where epithelial tumor cells acquire genetic and phenotypic characteristics of mesenchymal cells and these cells show enhanced invasive capacity and potentially stem cell characteristics [30,31].

However, Cav-1 may also directly affect tumor cells as transformation of NIH-3T3 cells with oncogenes such as H-Ras is associated with decreased Cav-1 expression and re-expression of Cav-1 can inhibit anchorage-independent growth of transformed NIH-3T3 cells. This finding, coupled with our observation that Cav-1 was significantly elevated in our DNA microarray comparing claudin-low mammary tumors to our primary, IGF-IR induced epithelial mammary tumors led us to examine the expression of Cav-1 in the tumor cells themselves. Claudin-low breast cancers are a subtype of human breast cancer characterized by low levels of epithelial genes and high levels of mesenchymal genes as well as genes involved in the immune response and those associated with stem cells [32,33].

In our MTB-IGFIR transgenic model [23,24], Cav-1 was found to be expressed in adipocytes and smooth muscle cells in the mammary gland but the cells of mammary tumors induced by IGF-IR overexpression expressed considerably less Cav-1. Most of the epithelial cells of the mammary tumors expressed little or no Cav-1 protein. However, there were small pockets of cells within some of the IGF-IR induced mammary tumors. Interestingly, these pockets of Cav-1 cells were in close proximity to cells within the tumor expressing high levels of cytokeratin 5 or cytokeratin 14 which were frequently were associated with ghost cells. In contrast, mammary tumors that develop following IGF-IR downregulation and acquire a claudin-low genotype express moderate to high levels of Cav-1 in the tumor cells themselves. This pattern of Cav-1 expression was maintained in mammary tumor cell lines derived from MTB-IGFIR transgenic tumors with epithelial or claudin-low characteristics. Therefore, Cav-1 levels were higher in claudin-low tumor cells than epithelial tumor cells.

This association of Cav-1 with claudin-low cells supports the findings of Bailey and Liu [34] who observed that induction of EMT in embryonic carcinoma cells or murine mammary epithelial cells was associated with elevated Cav-1 expression. In addition, Orlichenko et al. observed that decreased Cav-1 expression or function resulted in elevated E-cadherin levels in MDCK epithelial cells [35]. Moreover, Savage et al. [36] found that Cav-1 was expressed at high levels in basal-like and metaplastic breast tumors while Prat et al. [33] and Haakensen et al. [37] have observed higher levels of Cav-1 in human claudin-low breast tumors or cell lines.

These findings are all consistent with our findings since we found that IGF-IR-induced mammary tumors and murine mammary tumor cell lines 4T1 and RJ345 that expressed high levels of E-cadherin and low levels of mesenchymal genes also expressed low levels of Cav-1. In contrast, IGF-IR independent mammary tumors and the murine mammary tumor cell lines RJ348 and RM11A that expressed very low levels of E-cadherin and had gene expression patterns similar to claudin-low tumors also expressed considerable amounts of Cav-1. Therefore, our study supports the findings that claudin-low mammary tumors express more Cav-1 than epithelial mammary tumors. Cav-1 may be useful with other markers in identifying/distinguishing claudin-low human breast tumors.

In general, lung metastases expressed low levels of Cav-1 protein while normal lung alveolar cells expressed much higher levels of Cav-1. The alveolar expression of Cav-1 was not unexpected as Cav-1 has been reported to be expressed in type I pneumocytes [38]. This finding suggests that Cav-1 is not important in growth of tumors at secondary sites. Although we have not extensively characterized the lung metastases these lesions do appear to have an epithelial morphology and thus if the tumor cells in the metastases are primarily epithelial we would not expect them to express high levels of Cav-1. A search of the literature did not reveal any papers describing the expression of Cav-1 in breast metastatic lesions.

Our findings suggest that Cav-1 expression is dependent of the subtype of mammary tumor being investigated. It appears that epithelial mammary tumors express low levels of Cav-1 but mammary tumors that acquire claudin-low characteristics express Cav-1. Further investigations are required to determine whether Cav-1 can be used as a marker and/or a therapeutic target for claudin-low breast cancers.

## Competing interests

The authors declare that they have no competing interests.

## Authors' contributions

DET performed the western blot, immunofluorescence and RT-PCR for Cav1 while MDS sectioned all of the tissue, performed the histology and immunohistochemistry. RAM ran the project and wrote the manuscript. All authors read and approved the final manuscript.

## References

[B1] Scherer PE Okamoto T Chun M Nishimoto I Lodish HF Lisanti MP Identification, sequence, and expression of caveolin-2 defines a caveolin gene family Proc Natl Acad Sci USA 1996 93 131 135 10.1073/pnas.93.1.131 8552590 PMC40192

[B2] Kurzchalia TV Dupree P Parton RG Kellner R Virta H Lehnert M VIP21, a 21-kD membrane protein is an integral component of trans-Golgi-network-derived transport vesicles J Cell Biol 1992 118 1003 1014 10.1083/jcb.118.5.1003 1512286 PMC2289580

[B3] Tang Z Scherer PE Okamoto T Song K Chu C Kohtz DS Molecular cloning of caveolin-3, a novel member of the caveolin gene family expressed predominantly in muscle J Biol Chem 1996 271 2255 2261 10.1074/jbc.271.4.2255 8567687

[B4] Way M Parton RG M-caveolin, a muscle-specific caveolin-related protein FEBS Lett 1995 376 108 112 10.1016/0014-5793(95)01256-7 8521953

[B5] Witkiewicz AK Casimiro MC Dasgupta A Mercier I Wang C Bonuccelli G Towards a new "stromal-based" classification system for human breast cancer prognosis and therapy Cell Cycle 2009 8 1654 1658 10.4161/cc.8.11.8544 19448435

[B6] Witkiewicz AK Dasgupta A Sotgia F Mercier I Pestell RG Sabel M An absence of stromal caveolin-1 expression predicts early tumor recurrence and poor clinical outcome in human breast cancers Am J Pathol 2009 174 2023 2034 10.2353/ajpath.2009.080873 19411448 PMC2684168

[B7] Migneco G Whitaker-Menezes D Chiavarina B Castello-Cros R Pavlides S Pestell RG Glycolytic cancer associated fibroblasts promote breast cancer tumor growth, without a measurable increase in angiogenesis: Evidence for stromal-epithelial metabolic coupling Cell Cycle 2010 9 2412 2422 10.4161/cc.9.12.11989 20562527

[B8] Pavlides S Tsirigos A Vera I Flomenberg N Frank PG Casimiro MC Loss of stromal caveolin-1 leads to oxidative stress, mimics hypoxia and drives inflammation in the tumor microenvironment, conferring the "reverse Warburg effect": A transcriptional informatics analysis with validation Cell Cycle 2010 9 2201 2219 10.4161/cc.9.11.11848 20519932

[B9] Bonuccelli G Whitaker-Menezes D Castello-Cros R Pavlides S Pestell RG Fatatis A The reverse Warburg effect: glycolysis inhibitors prevent the tumor promoting effects of caveolin-1 deficient cancer associated fibroblasts Cell Cycle 2010 9 1960 1971 10.4161/cc.9.10.11601 20495363

[B10] Pavlides S Whitaker-Menezes D Castello-Cros R Flomenberg N Witkiewicz AK Frank PG The reverse Warburg effect: aerobic glycolysis in cancer associated fibroblasts and the tumor stroma Cell Cycle 2009 8 3984 4001 10.4161/cc.8.23.10238 19923890

[B11] Martinez-Outschoorn UE Balliet RM Rivadeneira DB Chiavarina B Pavlides S Wang C Oxidative stress in cancer associated fibroblasts drives tumor-stroma co-evolution: A new paradigm for understanding tumor metabolism, the field effect and genomic instability in cancer cells Cell Cycle 2010 9 3256 3276 10.4161/cc.9.16.12553 20814239 PMC3041164

[B12] Martinez-Outschoorn UE Trimmer C Lin Z Whitaker-Menezes D Chiavarina B Zhou J Autophagy in cancer associated fibroblasts promotes tumor cell survival: Role of hypoxia, HIF1 induction and NFkappaB activation in the tumor stromal microenvironment Cell Cycle 2010 9 3515 3533 10.4161/cc.9.17.12928 20855962 PMC3047617

[B13] Pavlides S Tsirigos A Migneco G Whitaker-Menezes D Chiavarina B Flomenberg N The autophagic tumor stroma model of cancer: Role of oxidative stress and ketone production in fueling tumor cell metabolism Cell Cycle 2010 9 3485 3505 10.4161/cc.9.17.12721 20861672 PMC3047615

[B14] Trimmer C Sotgia F Whitaker-Menezes D Balliet RM Eaton G Martinez-Outschoorn UE Caveolin-1 and mitochondrial SOD2 (MnSOD) function as tumor suppressors in the stromal microenvironment: a new genetically tractable model for human cancer associated fibroblasts Cancer Biol Ther 2011 11 383 394 10.4161/cbt.11.4.14101 21150282 PMC3047109

[B15] Sager R Sheng S Anisowicz A Sotiropoulou G Zou Z Stenman G RNA genetics of breast cancer: maspin as paradigm Cold Spring Harb Symp Quant Biol 1994 59 537 546 10.1101/SQB.1994.059.01.060 7587110

[B16] Lee SW Reimer CL Oh P Campbell DB Schnitzer JE Tumor cell growth inhibition by caveolin re-expression in human breast cancer cells Oncogene 1998 16 1391 1397 10.1038/sj.onc.1201661 9525738

[B17] Heiser LM Wang NJ Talcott CL Laderoute KR Knapp M Guan Y Integrated analysis of breast cancer cell lines reveals unique signaling pathways Genome Biol 2009 10 R31 10.1186/gb-2009-10-3-r31 19317917 PMC2691002

[B18] Elsheikh SE Green AR Rakha EA Samaka RM Ammar AA Powe D Caveolin 1 and Caveolin 2 are associated with breast cancer basal-like and triple-negative immunophenotype Br J Cancer 2008 99 327 334 10.1038/sj.bjc.6604463 18612310 PMC2480981

[B19] Razani B Lisanti MP Caveolin-deficient mice: insights into caveolar function human disease J Clin Invest 2001 108 1553 1561 11733547 10.1172/JCI14611 PMC201001

[B20] Yang G Timme TL Naruishi K Fujita T Fattah el MA Cao G Mice with cav-1 gene disruption have benign stromal lesions and compromised epithelial differentiation Exp Mol Pathol 2008 84 131 140 10.1016/j.yexmp.2007.08.004 18358473

[B21] Williams TM Sotgia F Lee H Hassan G Di VD Bonuccelli G Stromal and epithelial caveolin-1 both confer a protective effect against mammary hyperplasia and tumorigenesis: Caveolin-1 antagonizes cyclin D1 function in mammary epithelial cells Am J Pathol 2006 169 1784 1801 10.2353/ajpath.2006.060590 17071600 PMC1780215

[B22] Franks SE Campbell CI Barnett EF Siwicky MD Livingstone J Cory S Transgenic IGF-IR overexpression induces mammary tumors with basal-like characteristics while IGF-IR independent mammary tumors express a claudin-low gene signature Oncogene 2011 in press 10.1038/onc.2011.486 PMC3391665 22020329

[B23] Jones RA Campbell CI Gunther EJ Chodosh LA Petrik JJ Khokha R Transgenic overexpression of IGF-IR disrupts mammary ductal morphogenesis and induces tumor formation Oncogene 2007 26 1636 1644 10.1038/sj.onc.1209955 16953219

[B24] Jones RA Campbell CI Wood GA Petrik JJ Moorehead RA Reversibility and recurrence of IGF-IR-induced mammary tumors Oncogene 2008 13 407 413 10.1038/onc.2009.79 19377512

[B25] Campbell CI Thompson DE Siwicky MD Moorehead RA Murine mammary tumor cells with a claudin-low genotype Cancer Cell Int 2011 11 28 10.1186/1475-2867-11-28 21846397 PMC3170246

[B26] Jones RA Campbell CI Petrik JJ Moorehead RA Characterization of a novel primary mammary tumor cell line reveals that cyclin D1 is regulated by the type I insulin-like growth factor receptor Mol Cancer Res 2008 6 819 828 10.1158/1541-7786.MCR-07-2157 18505926

[B27] Aslakson CJ Miller FR Selective events in the metastatic process defined by analysis of the sequential dissemination of subpopulations of a mouse mammary tumor Cancer Res 1992 52 1399 1405 1540948

[B28] Chiavarina B Whitaker-Menezes D Migneco G Martinez-Outschoorn UE Pavlides S Howell A HIF1-alpha functions as a tumor promoter in cancer associated fibroblasts, and as a tumor suppressor in breast cancer cells: Autophagy drives compartment-specific oncogenesis Cell Cycle 2010 9 3534 3551 10.4161/cc.9.17.12908 20864819 PMC3047618

[B29] Sotgia F Del GF Casimiro MC Bonuccelli G Mercier I Whitaker-Menezes D Caveolin-1-/- null mammary stromal fibroblasts share characteristics with human breast cancer-associated fibroblasts Am J Pathol 2009 174 746 761 10.2353/ajpath.2009.080658 19234134 PMC2665737

[B30] Mani SA Guo W Liao MJ Eaton EN Ayyanan A Zhou AY The epithelial-mesenchymal transition generates cells with properties of stem cells Cell 2008 133 704 715 10.1016/j.cell.2008.03.027 18485877 PMC2728032

[B31] Hollier BG Evans K Mani SA The epithelial-to-mesenchymal transition and cancer stem cells: a coalition against cancer therapies J Mammary Gland Biol Neoplasia 2009 14 29 43 10.1007/s10911-009-9110-3 19242781

[B32] Herschkowitz JI Simin K Weigman VJ Mikaelian I Usary J Hu Z Identification of conserved gene expression features between murine mammary carcinoma models and human breast tumors Genome Biol 2007 8 R76 10.1186/gb-2007-8-5-r76 17493263 PMC1929138

[B33] Prat A Parker JS Karginova O Fan C Livasy C Herschkowitz JI Phenotypic and molecular characterization of the claudin-low intrinsic subtype of breast cancer Breast Cancer Res 2010 12 R68 10.1186/bcr2635 20813035 PMC3096954

[B34] Bailey KM Liu J Caveolin-1 up-regulation during epithelial to mesenchymal transition is mediated by focal adhesion kinase J Biol Chem 2008 283 13714 13724 10.1074/jbc.M709329200 18332144 PMC2376249

[B35] Orlichenko L Weller SG Cao H Krueger EW Awoniyi M Beznoussenko G Caveolae mediate growth factor-induced disassembly of adherens junctions to support tumor cell dissociation Mol Biol Cell 2009 20 4140 4152 10.1091/mbc.E08-10-1043 19641024 PMC2754928

[B36] Savage K Lambros MB Robertson D Jones RL Jones C Mackay A Caveolin 1 is overexpressed and amplified in a subset of basal-like and metaplastic breast carcinomas: a morphologic, ultrastructural, immunohistochemical, and in situ hybridization analysis Clin Cancer Res 2007 13 90 101 10.1158/1078-0432.CCR-06-1371 17200343

[B37] Haakensen VD Lingjaerde OC Luders T Riis M Prat A Troester MA Gene expression profiles of breast biopsies from healthy women identify a group with claudin-low features BMC Med Genomics 2011 4 77 10.1186/1755-8794-4-77 22044755 PMC3216859

[B38] Glenney JR Jr Soppet D: Sequence and expression of caveolin, a protein component of caveolae plasma membrane domains phosphorylated on tyrosine in Rous sarcoma virus-transformed fibroblasts Proc Natl Acad Sci USA 1992 89 10517 10521 10.1073/pnas.89.21.10517 1279683 PMC50370

